# Recording interventionalists’ occupational dose by clinical procedure: a novel approach to radiation safety

**DOI:** 10.1186/s42155-026-00658-y

**Published:** 2026-02-28

**Authors:** Roberto M. Sanchez, Eliseo Vaño Carruana, José Miguel Fernández Soto

**Affiliations:** 1 Medical Physics Service, Hospital Clínico San Carlos and IdISSC, Madrid, Spain; 2 Present Address: Radiology Department, Medicine Faculty, Universidad Complutense de Madrid, Madrid, Spain

**Keywords:** Occupational dosimetry, Interventional radiology, Patient doses, Alerts, Optimisation

## Abstract

**Objectives:**

The number of fluoroscopy-guided interventional procedures has increased in recent years due to their significant patient benefits. However, occupational radiation risks for interventionalists remain among the highest in medical practices. Electronic personal dosimeters can measure occupational doses for individual procedures, enabling immediate optimisation actions. An information system that effectively aggregates these records proves to be a powerful tool for this optimisation. Furthermore, implementing alerts to identify abnormal occupational doses is a practical method to improve radiation protection for interventionists.

**Methods:**

Electronic dosimeters were linked with the X-ray system to record both the occupational and patient doses on a per-procedure basis. The information, stored in a central database, is used to compute dose indicators and trigger alerts that warn operators about specific areas requiring optimisation.

**Results:**

The results from the first 9 months of 2024 were analysed, covering 3 interventional cardiology rooms and 2 interventional radiology rooms, with 19 interventionalists (8 cardiologists and 11 radiologists) performing a total of 3373 procedures. The initial alerts were configured on the basis of regulatory dose limits and the third quartile of occupational dose per procedure, as well as the ratio of occupational dose to kerma area product per procedure and the ratio between occupational dose and the C-arm measured dose.

**Conclusions:**

These alerts, along with personal dose data and other dosimetry information, are accessible to interventionalists and medical physics experts. This information system provides detailed, structured occupational dose data necessary to implement targeted optimisation measures.

**Graphical Abstract:**

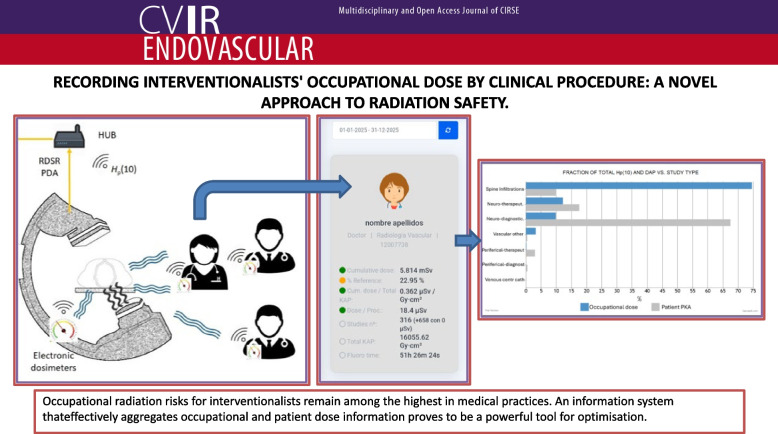

## Introduction

The number of fluoroscopy-guided interventional procedures (FGIP) is increasing not only in interventional cardiology and radiology but also in many other medical surgical specialties [1, 2]. Occupational radiation risks for interventionists are among the highest in medical practices [3, 4]. The European dose limit on the equivalent dose for the lens of the eye of 20 mSv per year is an additional challenge in radiation protection (RP) for interventionists [5, 6]. While the industry is improving X-ray systems to reduce patient and staff doses while maintaining image quality [7], some interventionalists are still at risk of exceeding the eye lens dose limit [2].

In recent years, dose management systems (DMS) and electronic personal dosimeters are helping with the integration of patient and occupational protection, as recommended by the ICRP and others [2, 8, 9]. The integration of clinical procedure details with occupational dose data provides valuable insights into the origin of occupational scatter radiation, facilitating enhanced optimisation strategies.

The use of two personal dosimeters (recognised as the “double-dosimeter approach”), one under the protective apron and another unshielded, over the apron, reference (ambient) dosimeter at the C-arm, has been recommended by the ICRP for optimisation of occupational protection during interventional procedures [2]. Double personal dosimetry allows a good approach to estimate the effective dose and a reasonable estimate for the eyes estimated by the “over apron” dosimeter [2, 9]. Several studies have analysed occupational dose measurements at the collar level or at the chest over the apron, compared with doses measured outside protective eyewear, showing a low but significant correlation, with average ratios eye/collar or eye/chest of 0.75 and quartiles of 0.5, 0.6, and 0.9 [10, 11].

Including the kerma area product delivered by the C-arm together with the over-apron *H*_p_(10) readings can provide an indication of the proper use of distance to the patient or other protection means, such as a ceiling-suspended screen. The reference (ambient) dosimeter positioned on the C-arm can also be a good control for the level of scatter radiation during FGIP [2].

Electronic personal dosimeters have some limitations (such as the accuracy for high-dose rates, angular and energy dependence, etc.), but they have other advantages, such as the possibility of being integrated into the DMS with occupational dose measurements for individual procedures and estimating lens doses from their over the apron readings [8, 9, 12–15].

It is usually expected that more complex procedures (with high patient dose values) involve higher occupational doses, but this is not always the case, and sometimes simple procedures, with low values of patient doses, involve high occupational doses due to the lack of proper use of RP tools [16]. The use of “alerts” for interventionists, generated automatically, may help improve occupational RP.

The European Directive 2013/59/EURATOM [6] requires to ensure justification and optimisation of the radiological procedures. For individual optimisation, especially for interventional procedures, patient and occupational doses should be part of the global optimisation process [17, 18].

This paper presents the experience gained from using DOSIM2, a prototype for occupational dose information system based on electronic dosimetry records structured on a procedure-by-procedure basis. The results during the first 9 months of 2024 in a large university hospital focused on a group of interventionists working in cardiology and radiology. Furthermore, we propose the criteria used to optimise occupational protection, which consider not only occupational dose values but also patient dose indicators and clinical information.

## Materials and methods

**Fig. 1:**
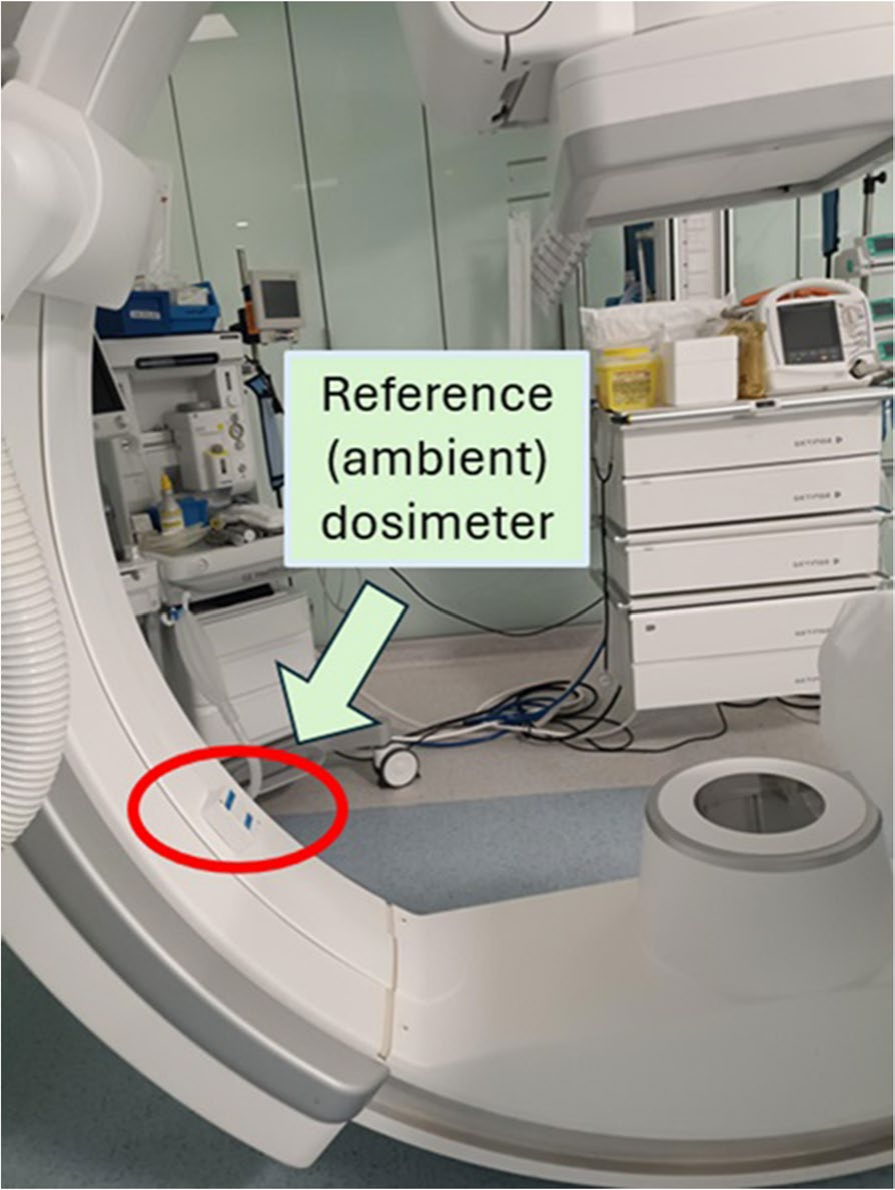
Reference (ambient) dosimeter at the C-arm

In addition to the existing DMS at San Carlos University Hospital DOLQA (by DOLQA systems) [9], a dedicated software package was produced for easy access to occupational doses in short periods of time, together with the values registered of patient dose indicators such as the kerma area product (KAP) of the interventional procedures and by the ambient dosimeter at the C-arm (Fig. 1).

The DOSIM2 offers information on personal occupational doses together with patient dose indicators and information about the clinical procedures, in selected periods of time, together with graphs containing doses from other (anonymized) colleagues working in the same catheterisation laboratory. The new software is an advanced update of the DOSIM, which did not include the patient dose information [19].

The interventional radiologists and cardiologists involved use electronic personal dosimeters over the apron (RaySafe model i3, www.raysafe.com) in addition to the mandatory passive dosimetry required by the National Regulatory Authority.

The system takes advantage of the DoseAware Xtend (Philips Healthcare, the Netherlands) consisting in a dedicated solution that provides real-time readings on occupational dose for Philips Azurion interventional systems. The RaySafe i3 dosimeters send the H_p_(10) readings wirelessly in real time to a hub installed in the catheterisation room. This hub is also connected to the modality and stores the measured occupational dose structured at irradiation event level in an occupational dose structured report, which is sent to the DOSIM2 system when the patient is closed at the X-ray system [9]. The system also gathers the patient radiation dose structured report from the X-ray unit, which is linked to the previous report using the “study instance unique identification” DICOM label. Then, DOSIM2 stores the occupational and patient dose information for each clinical procedure in a MySQL (Oracle, USA) database.

We use the term “occupational dose” when referring to the personal dose equivalent (PDE) *H*_p_(10) measured in mSv (or µSv) by the electronic dosimeters. Following the recommendations mentioned in the introduction, we use the PDE as surrogate for eye lens dose.

The performance of the electronic dosimeters has been tested by the Medical Physics Service at San Carlos Hospital to ensure their good function and range of operation, including a periodic validation with a comparison with a calibrated electronic dosimeter.

### Information available for interventionists

The main information is directly presented by the DOSIM2 system to users by an HTTP service powered by an Apache server (https://apache.org) through a web navigator that takes records from the database and computes—for the user selected period of time—some pieces of information, including the cumulative occupational dose measured by the electronic dosimeter, the number of procedures in which the cardiologist or radiologist has been involved, the average ratio between the PDE and the KAP in µSv/(Gy·cm^2^) for the procedures involved, the average ratio between the PDE and the dose registered by the reference ambient dosimeter (in %), and the total KAP for all the procedures involved.

**Fig. 2:**
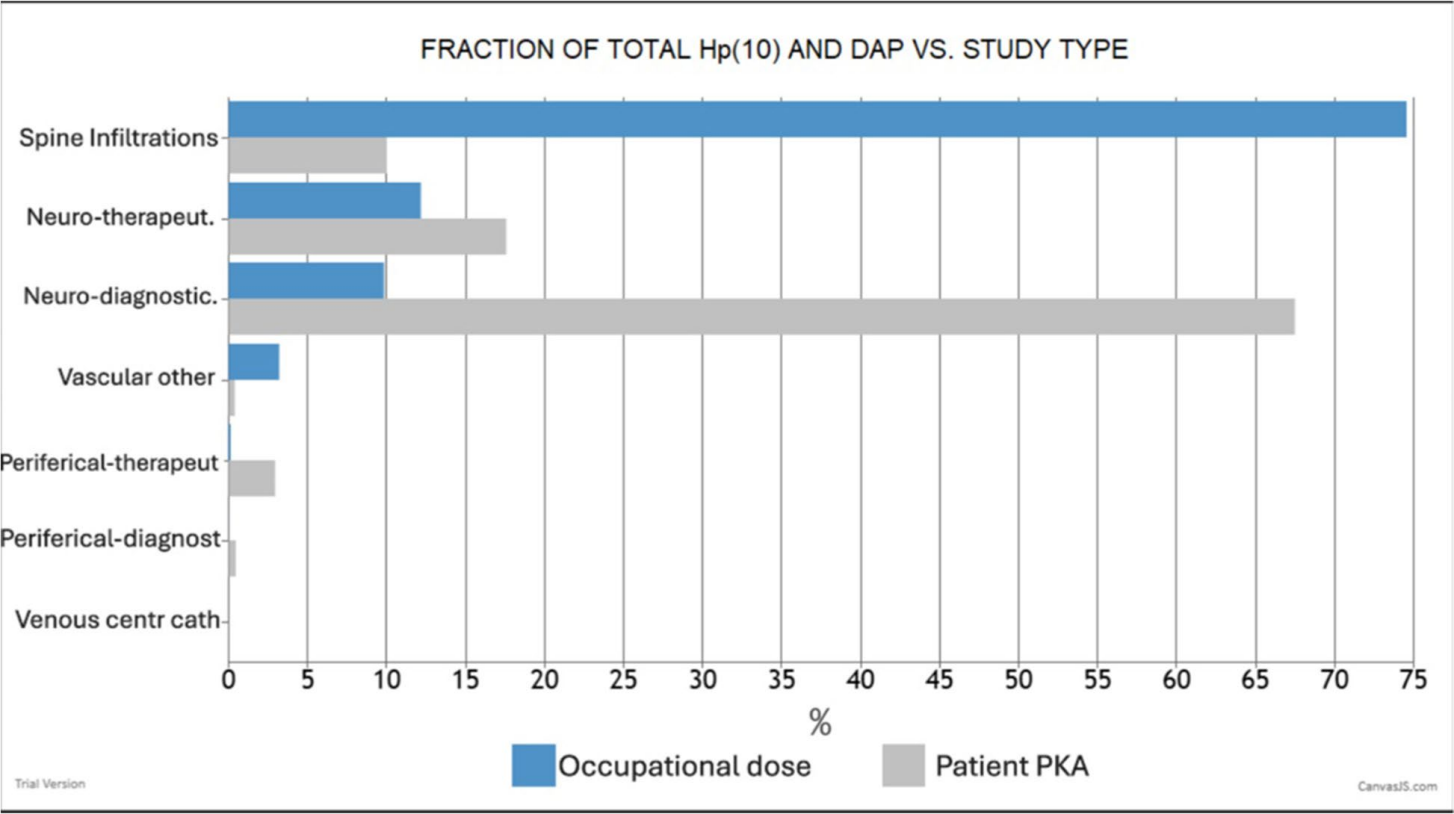
Example of a graph available in DOSIM2 for a neuroradiologist. Personal dose equivalent *H*_p_(10), measured on the apron in blue and the KAP in grey (equivalent to the DAP, dose area product, in the figure) for different procedures

On another screen (see Fig. 2), the following information is also available concerning the procedures or groups of procedures carried out by the interventionist: the number of procedures in that group, the percentage of the total occupational dose involved in those procedures, and the percentage of the total KAP for those procedures. This information is especially relevant for optimisation. This allows the identification of the group of procedures involving the highest occupational doses, which, together with the patient doses delivered in those procedures, can be used to assess how well professionals protect themselves when performing different procedure types.

The minimum user selected temporal resolution is 1 day; therefore, all displayed quantities are accumulated or averaged in this period. However, medical physicists in charge of the radiation protection and other authorised users have access to more detailed information, including the possibility of downloading the raw data in Excel sheets and having access to the global information available in DOSIM2, for all the operators and interventional rooms in the hospital network. This information comprises the following (for each procedure): cumulative doses for the reference (ambient) dosimeter in each room, the identification dosimeter number, and the total KAP (and fluoroscopy time) for the procedures completed in the rooms. It also differentiates the occupational dose recorded during fluoroscopy and digital acquisition (cine, DSA, road map, CBCT, etc.).

### Alerts for optimisation (using green, yellow, and red colours)

Optimisation alerts can be produced not only for abnormal (high) personal occupational dose values (compared with the lens dose limits and the 3rd quartile of the procedural dose) but also for the ratio of the operator dose to the patient dose (KAP) or to the dose values measured by the reference (ambient) dosimeter. These last two alerts may indicate the need to improve personal protection via the use of a ceiling-mounted screen, stepping back to increase distance during fluoroscopy or other protection strategies.

Personal occupational doses in a selected period may increase with the number and type of interventional procedures (especially their complexity). Increased filtration in the X-ray beam may reduce patient doses but may increase the amount and energy of scatter radiation, resulting in increased occupational radiation risk [20].

For the previously defined indicators shown for the time interval selected by the user, the suggested initial alerts (requiring corrective actions in some cases) are as follows:

- Alert 1: Cumulative PDE in mSv measured over the apron for the selected period.
- Alert 2: The average PDE (measured over the apron) per clinical procedure in µSv.
- Alert 3: Average ratio between the PDE (over the apron) and patient dose (KAP value) in µSv/(Gy·cm^2^).
- Alert 4: Average ratio between the PDE (over the apron) and the value measured by the C-arm reference dosimeter.

The four alert indicators are presented by the DOSIM2 system with three colours: green for values below the first notification, yellow for values between the 1 st and 2nd notification values, and red if the indicators are higher than the 2nd notification value.

For alert 1 (cumulative dose), the notification values were defined based on the investigation level and on the eye lens dose limit established in the national regulation. Therefore, for this alert, the 1 st and 2nd notification values are calculated, respectively, as the fractions—over the user selected period—of 6 mSv/year and 20 mSv/year. For example, if the user selects 2-month period in the system, the 1 st notification value is 1 mSv, and the 2nd notification value is 3.33 mSv. This alert aims to help keep cumulative doses under regulatory limits.

For alerts 2 (dose per procedure), 3 (µSv/Gy·cm^2^), and 4 (ratio PDE/C-arm), no recommendations were found in the literature. Because these alerts are intended for optimisation purposes, we adopted a criterion based on the statistical results obtained in our hospital. Consequently, the 1 st and 2nd notification values, respectively, were defined as the 3rd quartile and two times the 3rd quartile of our analysed sample. The analysed sample corresponds to the initial 9 months of 2024, with the experience of the new X-ray systems installed at the hospital at the end of 2023. These trigger values were selected to obtain an affordable number of alerts to keep alive the process of optimisation. In any case, the system administrator can modify the notification values and adapt them to their local characteristics.

## Results and discussion

Table 1 presents a summary of the results obtained from January to September 2024 in three laboratories performing all the cardiac interventional procedures (except the electrophysiology procedures) and two laboratories managing all the interventional radiology and neuroradiology procedures in the hospital.

**Table 1:** Median (interquartile range) and 3rd quartile values obtained from 19 interventionists over 9 months in 3 rooms for cardiology and 2 rooms for radiology. The proposed local alerts are based on the 3rd quartile values. The alerts for µSv/procedure and µSv/(Gy·cm^2^) were rounded to 25 and 0.50, respectively (see Fig. 3)

Procedures during the first 9 months of 2024 (total 3373)					
**Cardiology (8 interventionists) and 1088 procedures**					
Median (IQR)	Procedures/interventionist	mSv in 9 months	µSv/procedure	% reference	µSv/(Gy·cm^2^)
	150 (44)	2.50 (1.0)	15.3 (4.1)	10.9 (8.7)	0.24 (0.09)
**3rd quartile**	**160**	**2.89**	**18.0**	**14.4**	**0.30**
**Interventional radiology (11 interventionists) and 2285 procedures**					
Median (IQR)	Procedures/interventionist	mSv in 9 months	µSv/procedure	% reference	µSv/(Gy·cm^2^)
	211 (161)	3.28 (1.2)	17.2 (10)	16.8 (11.2)	0.31 (0.25)
**3rd quartile**	**277**	**3.70**	**21.1**	**22.5**	**0.44**

A total of 19 interventionists, 8 cardiologists and 11 radiologists with electronic dosimeters, were involved. Personal occupational doses were managed together with patient doses and the values of the reference (ambient) dosimeters for the procedures. The data collected by the DOSIM 2 system included a total of 3373 procedures (1088 in cardiology and 2285 in radiology).

The alerts 3 and 4 (the ratio between the PDE over the apron and patient dose (KAP value) in µSv/(Gy·cm^2^) and the ratio between the PDE and the value measured by the C-arm reference dosimeter) may have different relevance for cardiology and radiology, as well as simple and complex procedures, regarding the access route and the proper use of the ceiling-suspended screen (which is more difficult for some procedures in radiology than in cardiology).

**Fig. 3:**
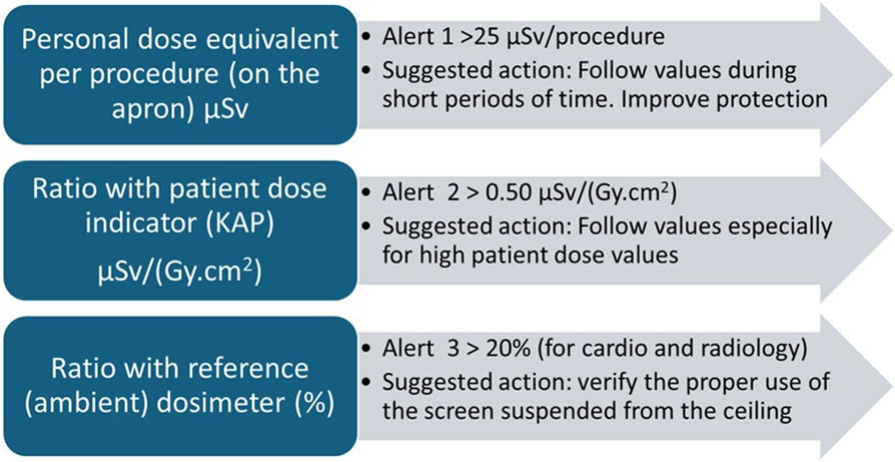
Summary of the suggested initial alerts to optimise occupational protection for interventional procedures in cardiology and radiology

The initial “alert” values adopted in the DOSIM2 system are summarised in Table 1 and Fig. 3. With these local alerts, it is possible to suggest optimisation actions to reduce occupational doses. These values should be periodically updated in the future if relevant changes occur (e.g. derived from more complex procedures or implementations of new interventional techniques).

Note that the 3rd quartile for the percentage of occupational dose compared with the reference dosimeter is higher for radiology than for cardiology. This is probably due to the large number of procedures in radiology without the possibility of using protective ceiling-suspended screens.

### Ratio between occupational dose and patient dose

One of the suggested alerts is the ratio between occupational doses (measured over the apron) and patient doses using the KAP (μSv/(Gy·cm^2^)). The impact of patient doses on occupational doses is relevant but depends on many geometric factors (C-arm angles, collimation, etc.), technical factors (especially kV and X-ray beam filtration) [20], and, of course, the proper use of the ceiling-suspended screen. For samples subjected to several procedures, high values of this ratio may be representative of riskier procedures for the staff or improper use of the ceiling-suspended screen.

Other authors have reported values for ratio between occupational and patient dose, ranging from 0.2 to 5 µSv/Gy·cm^2^, depending on the type of procedure [21]. The ORAMED study reported an average value of 1 µSv/Gy·cm^2^ for both cardiology and radiology procedures [22]. More recent studies have reported values between 1 and 2 µSv/Gy·cm^2^ [23], 0.3 and 3 µSv/Gy·cm^2^ [13], or 0.6–1 µSv/Gy·cm^2^ [24], most of them measured for first operators. At our centre, we obtained a median value of 0.2 µSv/Gy·cm^2^ for interventional cardiology and 0.3 µSv/Gy·cm^2^ for interventional radiology (Table 1). These values were derived from procedures performed during the study period and include measurements from both first and second operators. They were also obtained after installing new X-ray systems with dose reduction technology which also can help to reduce occupational doses [25].

Figure 2 shows an example of one of the graphs available in DOSIM2 for a neuroradiologist, where the fraction of the total dose received by the professional and his/her patients is grafted for each procedure type. Spinal infiltrations contributed most significantly to the operator’s dose (75%) while delivering only 10% of the patients’ KAP. These procedures are frequent but relatively simple, with short fluoroscopy times. In contrast, diagnostic neuroradiological procedures accounted for 65% of the patients’ KAP but contributed less than 10% to the occupational dose. This suggests that the interventionist employed more effective radiation protection strategies during diagnostic neuroradiological procedures than during spinal infiltrations, where significant potential for optimisation exists. Several RP training sessions were organised periodically with interventionists and medical physicists to discuss different options for optimisation.

### Alert management and optimisation actions

Analysis of the data for the full year 2024 revealed that among 21 monitored interventionalists, 6 triggered first-level notifications (yellow), while none triggered second-level notifications (red). Four of these interventionists had cumulative doses below 6 mSv/year (the threshold for a first notification). All affected staff were informed during training sessions and personal interviews, where factors affecting occupational dose were analysed including the complexity of the different types of procedures and the operator’s skills to protect themselves from the scatter radiation beams. Suggestions were provided to improve the use of ceiling-suspended screens.

Two interventionalists triggered first-notification alerts for cumulative annual doses exceeding 6 mSv, with readings of 8.2 and 8.4 mSv (measured over the apron without protection). Although these cumulative doses remained below regulatory limits for the eye lenses—even without considering eyewear protection—the values were approximately double the average of the other interventionalists. Consequently, the Medical Physics Department identified a clear margin for optimization.

Analysis of the ratio between the PDE and the KAP showed that both individuals triggered first notifications (> 0.5 µSv/Gy·cm^2^), with values of 0.47 and 0.50 µSv/Gy·cm^2^. This indicator suggest that the positioning of the ceiling-suspended screen could be improved.

The distribution of these annual doses across different procedure types showed that for both interventionalists, 17% of their PDE was received during biliary drainages, despite these procedures accounting for only 2.5% of the total KAP. During these procedures, the PDE/KAP ratio was approximately 3–4 µSv/Gy·cm^2^—three and four times higher than the second notification (red) threshold. These results were discussed with the practitioners to identify corrective actions, such as optimising the use of the ceiling-suspended screen or stepping back during fluoroscopy.

Comparing the evolution of these two workers in 2025 shows that one maintained similar indicators, while the other considerably reduced most metrics. This improvement, shown in Table 2, was made possible by the worker’s commitment to personal protection and the valuable information provided by the monitoring system.

**Table 2:**
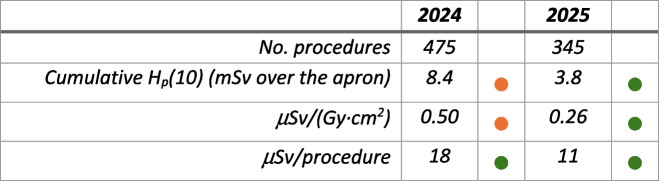
Indicators during the last 2 years for one interventional radiologist

### Limitations of the system and areas for improvement

This prototype, developed during a research collaboration with the Spanish regulatory authority, offers several advantages for optimising radiological protection for interventionalists and presents areas for improvement, such as the following:

- It is based on electronic dosimeters, which present limitations in their response to high-dose-rate pulsed radiation beams, as prevented by EURADOS [26]. This requires additional work from medical physics experts to ensure that the dosimeters are not working outside their range of operation. Manufacturers should improve their design using standards for pulsed radiation beams.
- A maximum loss of information of 5% of the total cumulative dose was measured when the reading of a dosimeter, stored in the internal dosimeter memory, was compared with the cumulative dose stored in the system. This loss was ascribed to communications failures. The system requires well-established communication links between its elements, i.e. dosimeters, hubs, X-rays, and the system itself, to ensure that there is no information loss, but also tools for connection monitoring. Dosimeters should provide information about their battery status or other issues to the system. Any disconnected element should be promptly detected to minimise and assess information loss.
- The tested prototype works with one manufacturer X-ray interventional machine (Philips) and one dosimeter manufacturer (RaySafe) only, who developed communications between its X-ray interventional units and the RaySafe DoseAware system. A communication standard for the occupational dose is needed to include all the required information for other dosimeters and X-ray manufacturers, including mobile C-arms used by surgeons in theatres.

## Conclusions

The prototype DOSIM2 offers detailed information to improve radiological protection for interventionalists, using personal-over-apron electronic dosimeters’ readings together with the patient dose information recorded for each clinical procedure. Medical physics experts can provide personalised advice about the specific types of procedures where protection should be improved.

A set of alerts was proposed for the cumulative dose, the average personal dose per procedure, the average ratio between occupational dose and patient dose or the average ratio between the occupational dose and the C-arm reference dosimeter. These alerts can also be seen by the operator wearing the dosimeter, fostering engagement in the optimisation process.

Official passive dosimetry is still required for regulatory purposes, but it is not enough to address precise and personalised optimisation actions for interventionalists, a collective operating in a high-radiation workplace.

## Data Availability

The datasets used and/or analysed during the current study are available from the corresponding author on reasonable request.

## Abbreviations

DMS: Dose management system
FGIP: Fluoroscopy-guided interventional procedures
KAP: Kerma area product
ODSR: Occupational dose structured reports
PDE: Personal dose equivalent
RP: Radiation protection
